# Screening Naturally Occurring Phenolic Antioxidants for Their Suitability as Additives to CHO Cell Culture Media Used to Produce Monoclonal Antibodies

**DOI:** 10.3390/antiox8060159

**Published:** 2019-06-03

**Authors:** Luis Toronjo-Urquiza, David C. James, Tibor Nagy, Robert J. Falconer

**Affiliations:** 1 Department of Chemical & Biological Engineering, ChELSI Institute, University of Sheffield, Sheffield S1 3JD, UK; ltoronjourquiza1@sheffield.ac.uk (L.T.-U.); d.c.james@sheffield.ac.uk (D.C.J.); 2 Fujifilm Diosynth Biotechnologies, Belasis Ave, Stockton-on-Tees, Billingham TS23 1LH, UK; tibor.nagy@fujifilm.com; 3 Department of Chemical Engineering, University of Adelaide, Adelaide, SA 5005, Australia

**Keywords:** flavan-3-ol, catechin, epicatechin, epigallocatechin gallate (EGCG), resveratrol, curcumin, monoclonal antibodies (mAb), immunoglobulin G (IgG), immunoglobulin, chemical chaperone

## Abstract

This study identified several antioxidants that could be used in Chinese hamster ovary (CHO)cell culture media and benefit monoclonal antibody production. The flavan-3-ols, catechin, epicatechin, epigallocatechin gallate and gallocatechin gallate all had no detrimental effect on cell viability at the concentrations tested, and they reduced the final viable cell count with a resulting rise in the cell specific productivity. The flavone, luteolin behave similarly to the flavan-3-ols. Resveratrol at 50 μM concentration resulted in the most pronounced reduction in viable cell density with minimal decrease in IgG synthesis and the largest increase in cell specific productivity. Low concentrations of α-tocopherol (35 μM) reduced viable cell density and raised cell specific productivity, but at higher concentration it had little additional effect. As high concentrations of α-tocopherol are not toxic to CHO cells, its addition as an anti-oxidant has great potential. Kaempferol up to 50 μM, curcumin up to 20 μM and piceid up to 100 μM showed little effect on growth or IgG synthesis and could be useful as antioxidants. Caffeic acid phenethyl ester was toxic to CHO cell and of no interest. Seven of the phenolic compounds tested are potential cell cycle inhibitors as well as having intrinsic antioxidant properties.

## 1. Introduction

Antioxidants are of potential benefit in the production of monoclonal antibodies (mAb) by Chinese hamster ovary (CHO) cells due to their capacity to reduce the damage by free radicals to the mAb and to the CHO cells in cell culture media. A range of small molecules of no direct nutritional value have been added to cell culture media to improve productivity or to protect the mAb being produced. It is the aim of this paper to assess whether a range of naturally occurring polyphenolic antioxidants can be added to the beneficial ingredients used in CHO cell culture media.

The productivity of mammalian cell culture has been the focus of considerable research and has resulted in marked improvements in reactor design and media composition. Early advances resulted in improved reactor design, enhanced feed strategies and improved media composition [1]. Researchers have also used mild hypothermia, hyperosmotic pressure and the addition of small molecules with no direct nutritional value to improve the cell specific productivity (*q_p_*) or reduce protein aggregation [2]. Temperature is a variable that impacts both the cell growth rate and the protein expression rate. By modulating temperature conditions can be provided to favour cell growth then protein synthesis optimising fed-batch or perfusion systems. Mild hypothermia (reduced temperature) has been used to slow cellular growth while allowing protein synthesis to continue, improving the *q_p_* values [3,4,5,6]. Temperature reduction can also reduce protein aggregation in a cell culture reactor [2]. Another method for modulating cell growth rates is by altering the osmotic pressure of the media. The negative effect caused by the hyperosmotic pressure conditions is counteracted by the addition of the osmolyte glycine betaine which achieves slower cellular growth and can in some circumstances improve the *q_p_* value [7,8]. The presence of low concentrations of glycine betaine does not interfere with cell growth or protein synthesis, its effect is solely due to modulating the osmotic pressure.

Some small molecules (often referred to as chemical chaperones) are capable of enhancing protein folding, improving protein solubility and slowing protein aggregation. The fatty acids, butyric acid, valeric acid, valproic acid and 4-phenylbutyric acid have been added to cell culture media [9,10,11,12,13]. Fatty acids have a hydrophobic end and a negatively charged end, so they are capable of hydrophobic interaction with a proteins hydrophobic surfaces displaying the charged end to the liquid-phase or an electrostatic interaction with positively charged side chains on the protein. The former interaction may enhance protein solubility while the second interaction could reduce solubility, so the effect is likely to be protein specific. These interactions are non-specific and will occur with the recombinant protein, other proteins and with the cells and their constituents. The fatty acids may also affect cell function. Sub-toxic doses might be cytostatic (which could increase the *q_p_* value) or induce apoptosis. Sodium butyrate slowed CHO cell growth and improved *q_p_* of recombinant tissue plasminogen activator production [9]. Valeric acid (pentanoic acid) produced higher product titres and increased *q_p_* for an unnamed recombinant protein in CHO cells [10]. Valproic acid (2-propylpentanoic acid) addition to cell culture media has also been reported to improve antibody expression [11,13]. 4-phenylbutyrate has also been used to minimise aggregation in protein expressed in CHO cells [12].

One family of chemicals that has received extensive research due to potential benefits to human health are the phenolic compounds, but research in their use as potential additives in cell culture media is limited [14,15,16]. Phenolic compounds are common in nature and play a range of roles, especially in vascular plants where they have been associated with antimicrobial activity, UV protection, antioxidant activity [17], and allelopathy [18]. Mammals have evolved in parallel with plants, and in many species plants form a major part in their diet. Herbivores and omnivores ingest significant quantities of phenolic compounds usually with little negative impact. In some cases, plant-derived phenolic compounds have been credited with a diverse range of benefits for the feeding animal. Polyphenols have had diverse medical attributes allocated to them, including the treatment and prevention of cancer and cardiovascular disease. They have also been suggested to be antiulcer, antithrombotic, anti-inflammatory, antiallergenic, anticoagulant, immune modulating, antimicrobial, vasodilatory, and analgesic [19]. Epigallocatechin gallate (EGCG), in particular, has been widely studied and it is claimed to regulate signal transduction pathways, transcription factors, DNA methylation, mitochondrial function, and autophagy [20]. The phenolic compound with obvious importance in human health is α-tocopherol, which is the form of vitamin E found in dietary supplements. Recent investigations into the beneficial properties of green tea, red wine, turmeric and leaf vegetables has resulted in extensive studies focusing on the flavan-3-ols, the flavonol kaempferol, the diarylheptanoid curcumin and the stilbene resveratrol.

**Figure 1:**
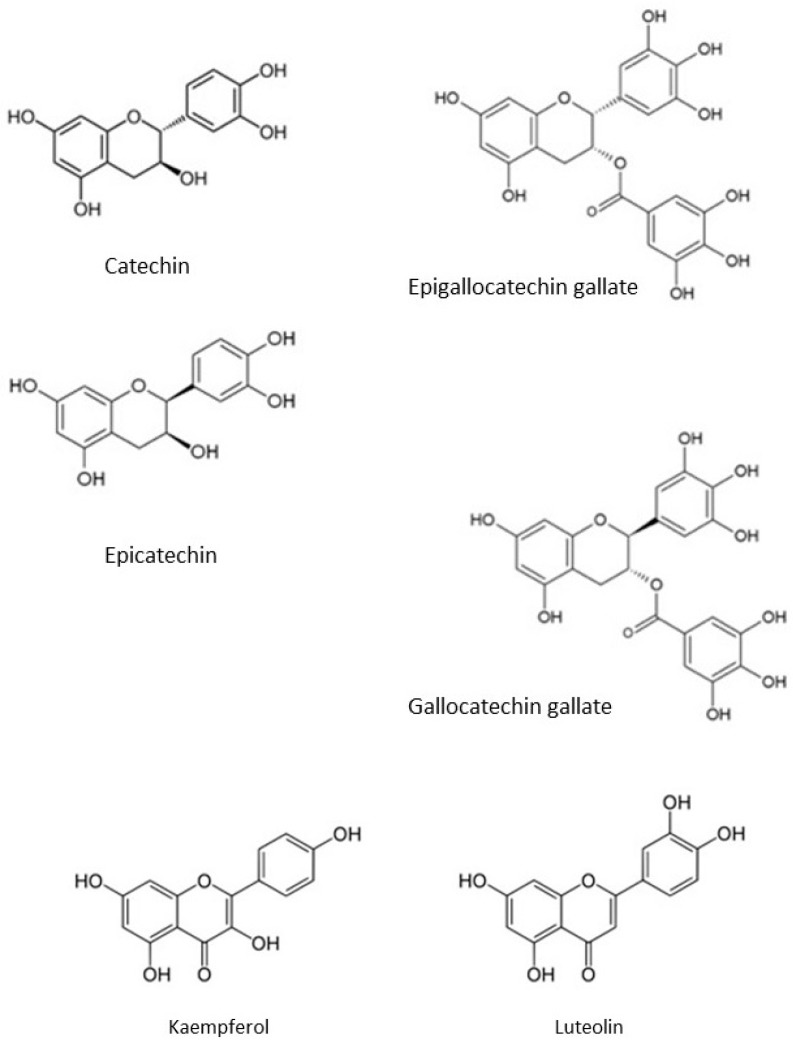

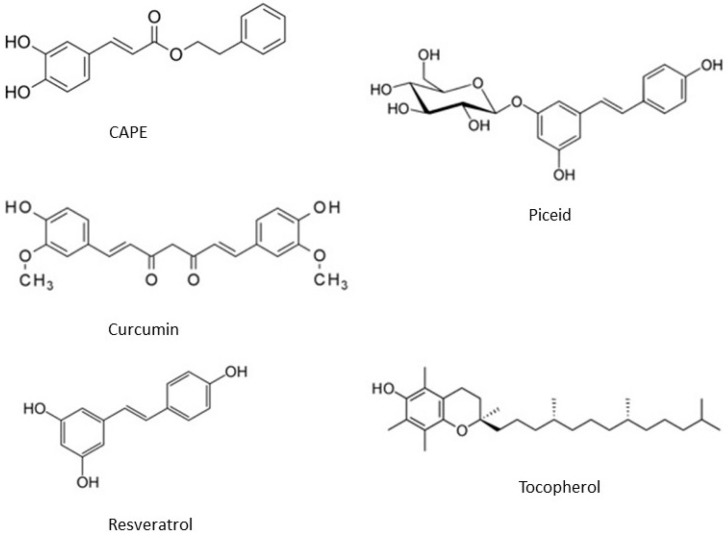
The structure of the flavan-3-ols; catechin, epicatechin, gallocatechin gallate and epigallocatechin gallate. The flavonol kaempferol and flavone luteolin. Caffeic acid phenethyl ester (CAPE), piceid, curcumin, resveratrol and α-tocopherol.

In this study, we screened a range of naturally occurring phenolic compounds found in the human diet for their effect on Chinese hamster ovary cells and the synthesis of IgG to detect whether they were potential candidates for cell cycle arrest, chemical chaperones or to provide antioxidant capabilities in cell culture media. The candidates included the flavan-3-ols, catechin, epicatechin, epigallocatechin gallate and gallocatechin gallate (Figure 1). These compounds are ubiquitous in vascular plants, and is found in products like cocoa, wine and tea. Kaempferol is a flavonol and is found in a wide range of vegetables and fruit. Apart from being an antioxidant it has also been linked to a wide range of beneficial health outcomes in humans. Luteolin is a flavone, is found in a range of leaf vegetables and is an antioxidant (Figure 1). Resveratrol is a stilbenoid and has two phenol groups connected by two double bonded carbons and is found in wine. It is an effective antioxidant and its potential health benefits have been widely studied. Piceid (also known as polydatin) is the glycoside form of resveratrol. Like resveratrol, it is found in a range of plants including grapes. Curcumin is a diarylheptanoid, it has two phenolic groups linked by a seven carbon chain. It is found in turmeric, where it imparts the yellow color and has antioxidant properties. Caffeic acid phenethyl ester (CAPE) is the ester of caffeic acid and phenethyl alcohol and is found in honey. α-tocopherol is one form of vitamin E and it is a fat soluble antioxidant found in a range of vegetable oils. The structures of CAPE, piceid, curcumin, resveratrol and α-tocopherol are shown in Figure 1. In screening candidates that are causing cell cycle arrest, it would be expected to reduce the viable cell count in the culture while having a less dramatic reduction in IgG synthesis. Chemical chaperones on the other hand, may increase the apparent IgG concentration by preventing aggregation. All candidates are capable of providing antioxidant capacity in cell culture media.

## 2. Materials and Methods

The following reagents were used during the process: dimethyl sulfoxide (DMSO), phosphate buffered saline (PBS), caffeine, 4-phenylbutyrate, glycine betaine, (±) catechin, (−) epicatechin, (−) gallocatechin gallate, epigallocatechin gallate, luteolin, kaempferol, resveratrol, piceid, caffeic acid phenethyl ester (CAPE), curcumin and α-tocopherol were supplied by Sigma-Aldrich (St. Louis, MI, USA). OptiCHO free serum media and l-glutamine were supplied by Thermo Fisher Scientific (Ulm, Germany). Further well known chaperon chemicals were also assessed as reference (Appendix B).

The cell line used was a stable recombinant DG44 CHO cell line that expressed an IgG monoclonal antibody, provided by Fujifilm Diosynth. This cell line was then left to grow in suspension in an OptiCHO™ free serum media in a 125 mL Erlenmeyer Flask (Corning, NY, USA) containing 33 mL of culture media, supplemented with 8 mM l-glutamine and 100 mg/L hygromycin B. Culture was maintained in a Infors HT Multitron cell shaking incubator (Bottmingen, Switzerland) set at 130 rpm with 5% CO_2_ modified atmosphere at 37 °C. Cells were seeded into new media at 0.2 × 10^6^ viable cells/mL every three to four days for a maximum of 16 passages prior to use.

### 2.1. 24-Well CHO Cell Growth and IgG Expression

The screening test was designed to estimate the concentration at which these chemicals become toxic on CHO cell lines and to determine whether the treatments affects IgG expression. Chemical treatments were carried in a Greiner bio-one Cellstar^®^ 24-well-plate (Stonehouse, UK), where 1 mL of exponentially growing cells were seeded at an initial viable cell density (VCD) of 0.2 × 10^6^ cells/mL. Wells were then treated at time 0 using a different range of concentrations for each chemical diluted in 5 μL of DMSO. The control wells were treated with the same volume of DMSO. Cells were grown for a period of three days in a static incubator (Infors HT) at 5% CO_2_ modified atmosphere and at a temperature of 37 °C.

### 2.2. Viable Cell Density, Viability and IgG Concentration Analysis

500 µL were used to measure viable cell density (VCD) and viability by cytometry using a Beckman-Coulter Vi-cell™ XR cell viability analyser (Indianapolis, IN, USA). 300 µL from the same samples were centrifuged for 10 min at 400 g, the supernatant collected and the IgG concentration measured using bio-layer interferometry using a Pall FortéBio Octet^®^ QK^e^ (Fremont, CA, USA).

Specific protein production (*q_p_*) was determined by dividing the total protein produced after three days incubation by the final VCD [21].

$$q_{p}\left(pg/cell\cdot day\right)=\frac{Final\;Recombinant\;titer\;Concentration\;(µg/mL)}{VCD\;\left(10^{6}\;viable\;cells/mL\right)\cdot days}$$

### 2.3. Statistical Analysis

A permutation test was performed to compare the differences between the control and the treatments for all the variables studied. *p*-values below 0.05 were considered significant. Data was analysed using R programming tool and the coin package [22].

## 3. Results

**Figure 2:**
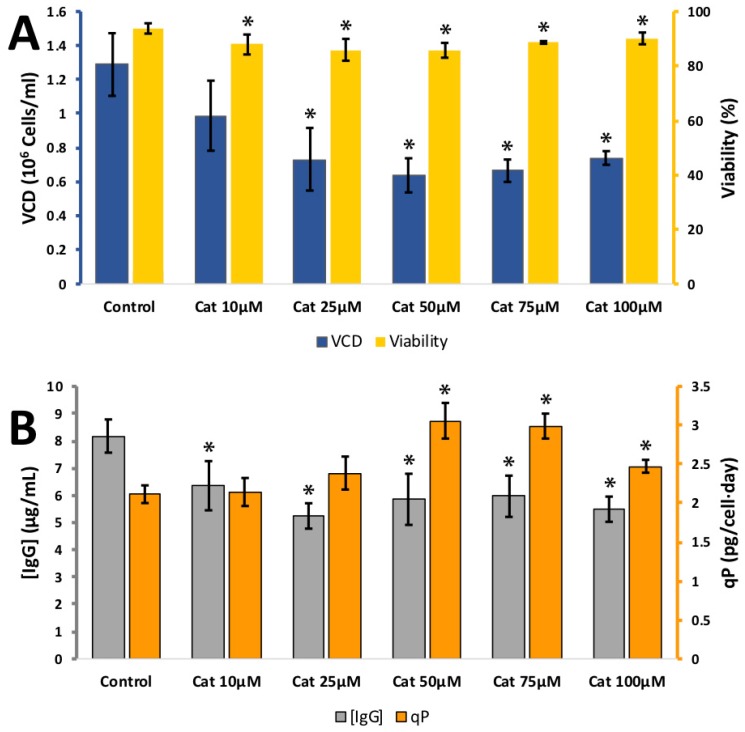
**A:** Effect of different catechin (Cat) concentrations on viable cell density (VCD) (blue) and viability (yellow) **B**: effect of different catechin concentrations (10–100 µM) IgG concentration (grey) and relative IgG production (qP) (orange) for CHO cells grown in 24-well plates. Each data point represents the average from a quadruplicate, and the error bars correspond to ±SD. * *p*-value < 0.05 for the treatment compared to the control.

**Figure A1:**
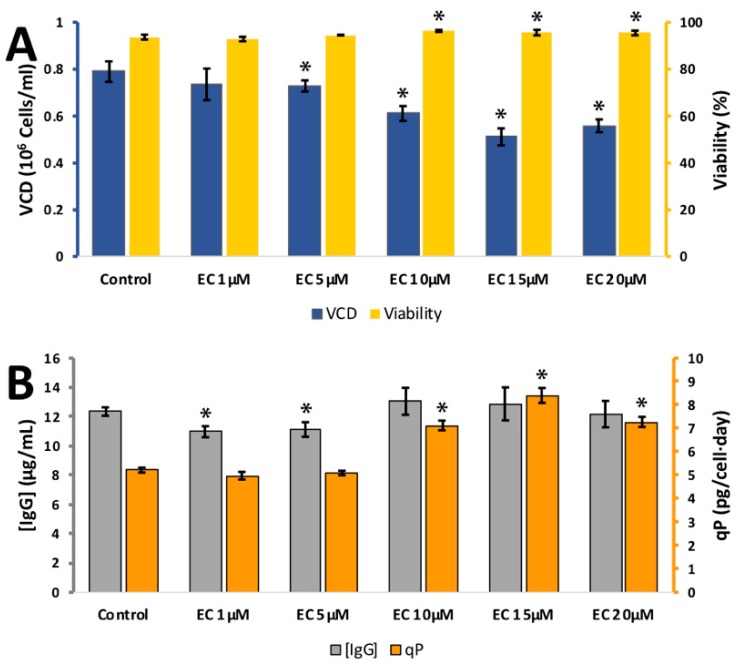
**A**: Effect of different epicatechin (EC) concentrations on viable cell density (VCD) (blue) and viability (yellow). **B**: effect of different epicatechin concentrations (10–100 µM) IgG concentration (grey) and relative IgG production (qP) (orange) for CHO cells grown in 24-well plates. Each data point represents the average from a quadruplicate, and the error bars correspond to ±SD. * *p*-value < 0.05 for the treatment compared to the control.

Catechin and epicatechin are isomers and only differ in the direction of the hydroxyl group on the carbon 3 on the dihydropyran (C) ring (see Figure 1). The effect of catechin on CHO cell viability, viable cell density (VCD) and IgG synthesis was tested at 10, 25, 50, 75 and 100 μM (Figure 2). Cell viability remained high over the whole concentration range. CHO cell growth was affected, the VCD dropped from 1.29 ± 0.18 × 10^6^ cells per mL at 0 μM catechin to 0.73 ± 0.18 × 10^6^ cells per mL for 25 μM and 0.64 ± 0.10 × 10^6^ cells per mL for 50 μM. Higher concentrations of catechin did not result in any further drop in VCD. IgG synthesis was reduced, but not to the same extent as the cell growth, final IgG concentration dropped from 8.2 ± 0.6 mg/L in the 0 μM catechin control to 6.3 ± 0.9 for 10 μM and 5.9 ± 0.8 for 50 μM, higher concentrations of catechin did not result in any further drop in IgG concentration. The calculated *q_p_* rose from 2.1 ± 0.1 pg/cell/day at 0 μM to 3.1 ± 0.2 pg/cell/day at 50 μM catechin. The effect of epicatechin on CHO cell viability, viable cell density (VCD) and IgG synthesis was tested at 1, 5, 10, 15 and 20 μM (Figure A1). Cell viability remained high over the whole concentration range. VCD dropped to 0.79 ± 0.04 × 10^6^ cells per mL for 0 μM and 0.56 ± 0.03 × 10^6^ cells per mL for 20 μM epicatechin. IgG synthesised remained stable over the concentration range tested. The calculated *q_p_* rose from 5.2 ± 0.1 pg/cell/day at 0 μM to 7.3 ± 0.2 pg/cell/day at 20 μM epicatechin. Unsurprisingly, the two isomers had a similar effect CHO cells.

**Figure 3:**
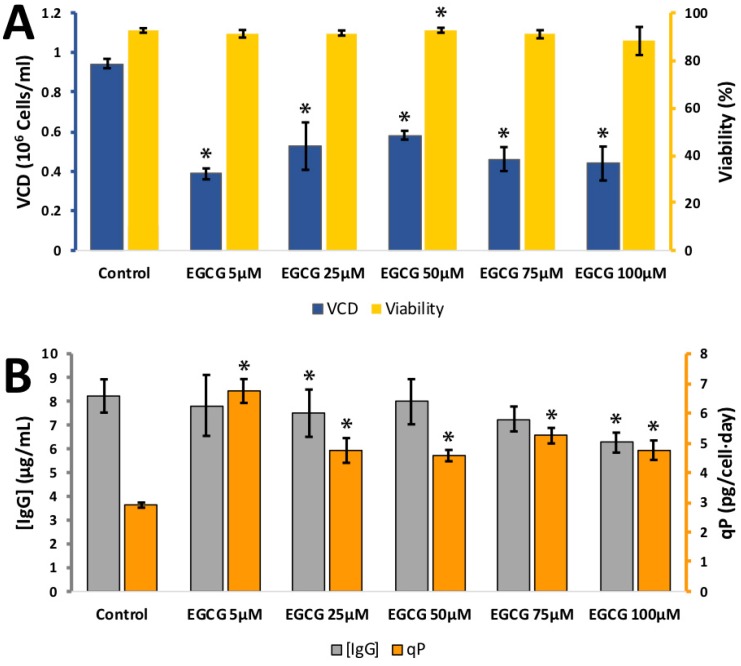
**A:** Effect of different epigallocatechin gallate (EGCG) concentrations on viable cell density (VCD) (blue) and viability (yellow) **B**: effect of different EGCG concentrations (10–100 µM) IgG concentration (grey) and relative IgG production (qP) (orange) for CHO cells grown in 24-well plates. Each data point represents the average from a quadruplicate, and the error bars correspond to ±SD. * *p*-value < 0.05 for the treatment compared to the control.

**Figure A2:**
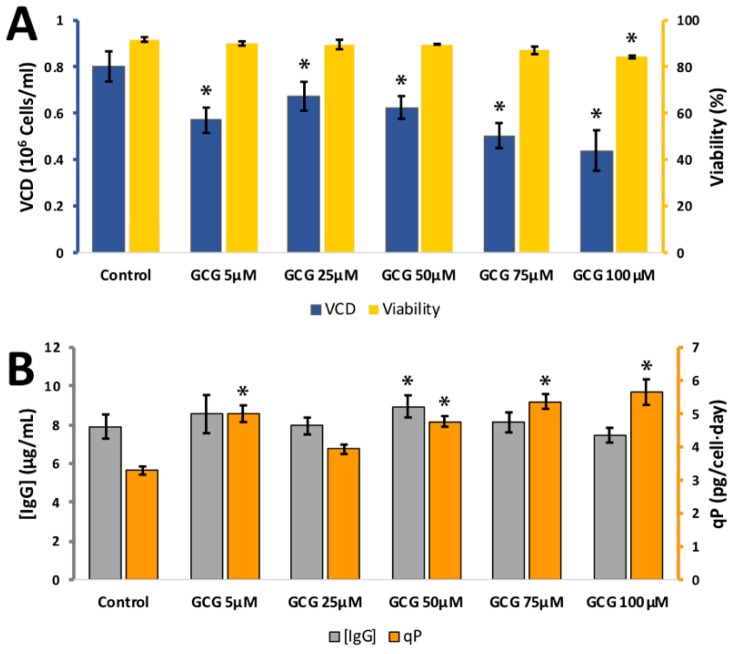
**A**: Effect of different gallocatechin gallate (GCG) concentrations on viable cell density (VCD) (blue) and viability (yellow). **B**: effect of different GCG concentrations (10–100 µM) IgG concentration (grey) and relative IgG production (qP) (orange) for CHO cells grown in 24-well plates. Each data point represents the average from a quadruplicate, and the error bars correspond to ±SD. * *p*-value < 0.05 for the treatment compared to the control.

Gallocatechin gallate (GCG) and epigallocatechin gallate (EGCG) are also isomers. They are gallic acid esters of gallocatechin and epigallocatechin, respectively. They differ in the direction of the ester bond on the carbon 3 on the dihydropyran (C) ring. GCG and EGCG are significantly larger than catechin and epicatechin, and have greater capacity to hydrogen bond than catechin and epicatechin. The effect of GCG on CHO cell viability, viable cell density (VCD) and IgG synthesis was tested at 5, 25, 50, 75 and 100 μM (Figure A2). Cell viability remained high over the whole concentration range. VCD dropped to 0.80 ± 0.06 × 10^6^ cells per mL for 0 μM and 0.44 ± 0.09 × 10^6^ cells per mL for 100 μM GCG. IgG synthesised remained stable over the concentration range tested. The calculated *q_p_* rose from 3.3 ± 0.1 pg/cell/day at 0 μM to 5.7 ± 0.4 pg/cell/day at 100 μM GCG. The effect of EGCG on CHO cell viability, viable cell density (VCD) and IgG synthesis was tested at 5, 25, 50, 75 and 100 μM (Figure 3). Cell viability remained high over the whole concentration range. VCD dropped from 0.94 ± 0.02 × 10^6^ cells per mL for 0 μM to 0.39 ± 0.03 × 10^6^ cells per mL for 5 μM, higher concentrations of EGCG did not result in any further drop in VCD. IgG synthesised dropped slightly over the concentration range in the experiment from 8.2 ± 0.7 mg/L in the 0 μM catechin control to 6.2 ± 0.4 mg/L for 100 μM EGCG. The calculated *q_p_* rose from 3.3 ± 0.1 pg/cell/day at 0 μM to 4.8 ± 0.3 pg/cell/day at 100 μM EGCG. Both GCG and EGCG had a marked impact on CHO VCD at the lowest concentration tested but no additional drop in VCD was observed at higher GCG or EGCG concentrations.

All of the flavan-3-ols had a similar impact on CHO cells and the synthesis of IgG being responsible for a reduction in VCD but a smaller effect on IgG synthesis resulting in a rise in *q_p_*. This suggests that the flavan-3-ols are all of potential use as a cell culture additive where a reduction cell growth is desirable and IgG synthesis is relatively unaffected.

**Figure 4:**
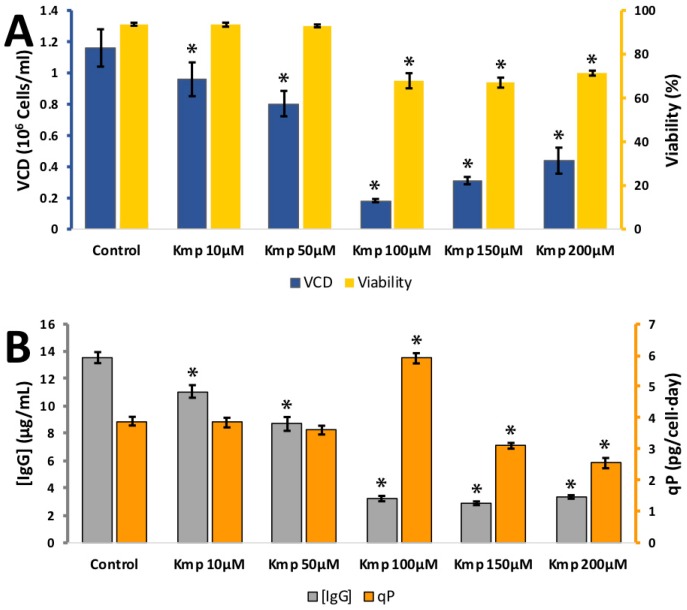
**A:** Effect of different kaempferol (Kmp) concentrations on viable cell density (VCD) (blue) and viability (yellow) **B**: effect of different kaempferol concentrations (10–100 µM) IgG concentration (grey) and relative IgG production (qP) (orange) for CHO cells grown in 24-well plates. Each data point represents the average from a quadruplicate, and the error bars correspond to ±SD. * *p*-value < 0.05 for the treatment compared to the control.

The flavonol, kaempferol’s effect on CHO cell growth and IgG synthesis was quite different to the flavan-3-ols. It was tested at 10, 50, 100, 150, and 200 μM kaempferol (Figure 4). Cell viability was unaffected between 0 and 50 μM, though there was a drop in VCD from 1.16 ± 0.12 × 10^6^ cells per mL at 0 μM to 0.80 ± 0.08 × 10^6^ cells per mL at 50 μM and a drop in IgG synthesis from 13.6 ± 0.4 mg/L at 0 μM to 8.7 ± 0.5 mg/L at 50 μM. The *q_p_* remained stable between 0 and 50 μM kaempferol. At 100 μM kaempferol viability and VCD dropped drastically, suggesting this dose was toxic to the cells. The evidence from this experiment suggest kaempferol could be used as a cell culture media additive up to a concentration of 50 μM, but there is no productivity benefit from its addition and could only be incorporated for its anti-oxidant properties.

**Figure 5:**
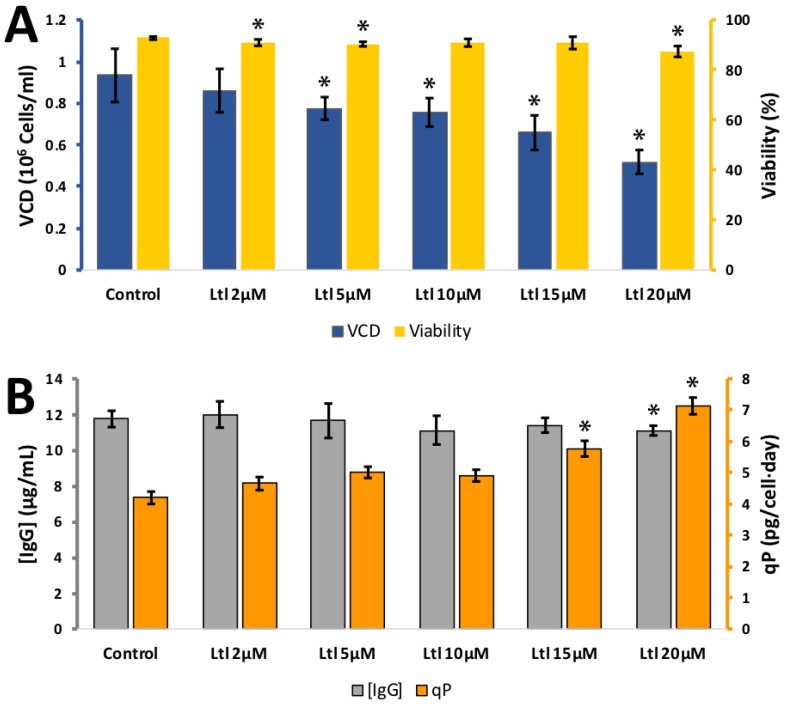
**A**: Effect of different luteolin (Ltl) concentrations on viable cell density (VCD) (blue) and viability (yellow) **B**: effect of different luteolin concentrations (10–100 µM) IgG concentration (grey) and relative IgG production (qP) (orange) for CHO cells grown in 24-well plates. Each data point represents the average from a quadruplicate, and the error bars correspond to ±SD. * *p*-value < 0.05 for the treatment compared to the control.

The flavone, luteolin was tested at 2, 5, 10, 15, and 20 μM (Figure 5). Cell viability remained high over the whole concentration range. VCD dropped to 0.80 ± 0.06 × 10^6^ cells per mL for 0 μM and 0.49 ± 0.01 × 10^6^ cells per mL for 20 μM. IgG synthesised remained stable over the concentration range tested. The calculated *q_p_* rose from 5.0 ± 0.1 pg/cell/day at 0 μM to 7.3 ± 0.2 pg/cell/day at 20 μM luteolin. This suggests that luteolin is of potential use as a cell culture additive where a reduction cell growth is desirable and IgG synthesis is relatively unaffected.

**Figure A3:**
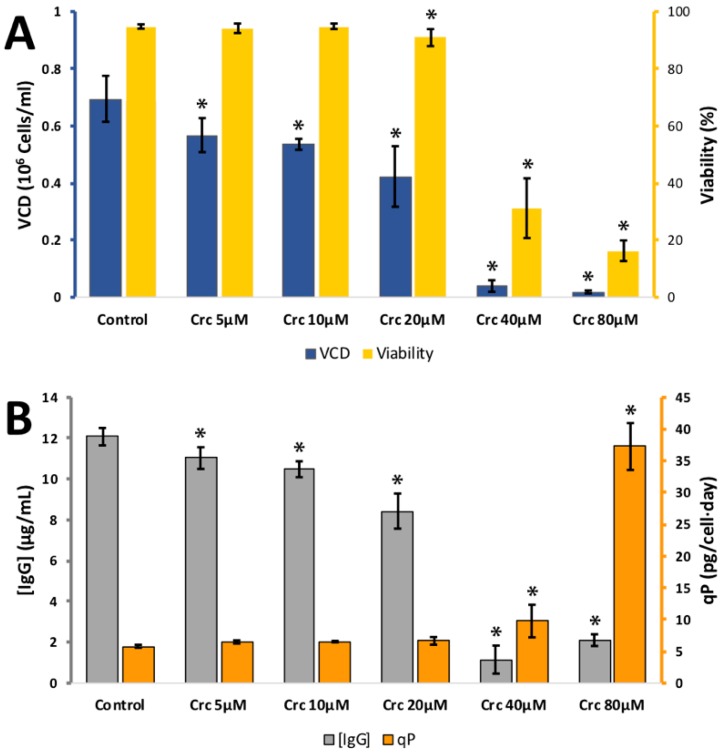
**A**: Effect of different curcumin (Crc) concentrations on viable cell density (VCD) (blue) and viability (yellow). **B**: effect of different curcumin concentrations (10–100 µM) IgG concentration (grey) and relative IgG production (qP) (orange) for CHO cells grown in 24-well plates. Each data point represents the average from a quadruplicate, and the error bars correspond to ±SD. * *p*-value < 0.05 for the treatment compared to the control.

CAPE was toxic to CHO cells even at the lowest concentration (2 μM) and was considered unsuitable as a cell culture media additive (Figure A3).

**Figure A4:**
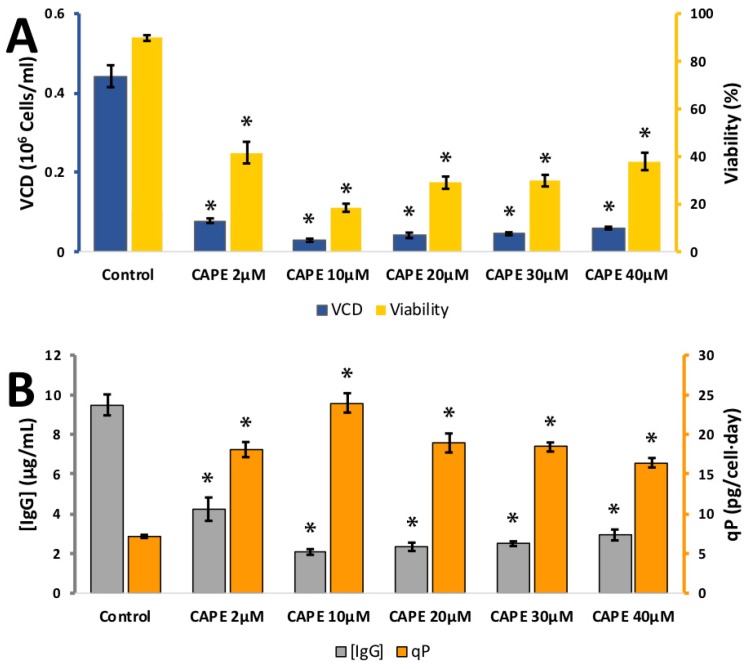
**A**: Effect of different caffeic acid phenethyl ester (CAPE) concentrations on viable cell density (VCD) (blue) and viability (yellow). **B**: effect of different CAPE concentrations (10–100 µM) IgG concentration (grey) and relative IgG production (qP) (orange) for CHO cells grown in 24-well plates. Each data point represents the average from a quadruplicate, and the error bars correspond to ±SD. * *p*-value < 0.05 for the treatment compared to the control.

Curcumin was tested at 5, 10, 20, 40 and 80 μM (Figure A4). Cell viability remained high between 0 and 20 μM, but lost viability at 40 μM. Between 0 and 20 μM curcumin there was a slight drop in VCD and IgG but the calculated *q_p_* remained stable. Curcumin could be added to media up to a concentration of 20 μM if its anti-oxidant properties were desired but are unlikely to provide any benefit in raising the *q_p_* value.

**Figure 6:**
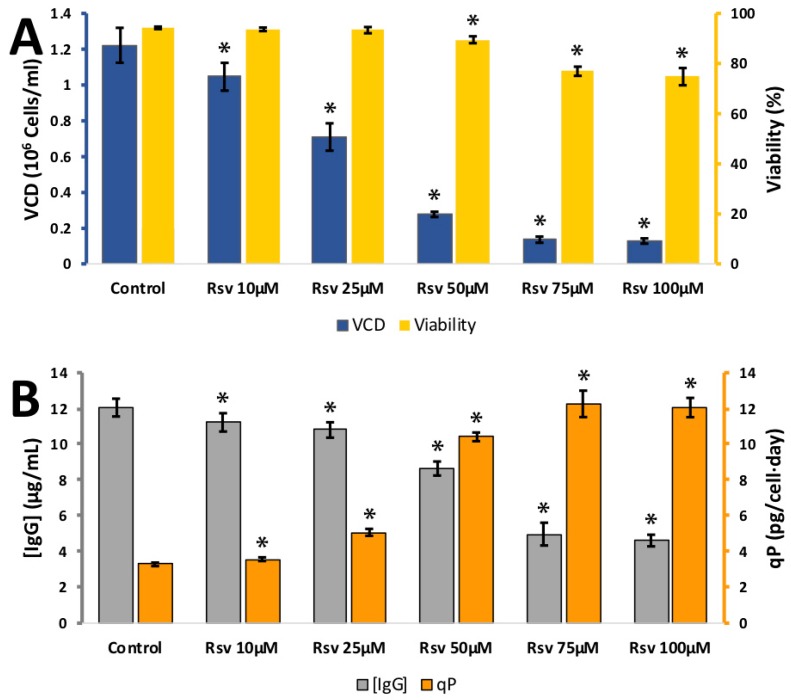
**A**: Effect of different resveratrol (Rsv) concentrations on viable cell density (VCD) (blue) and viability (yellow) **B**: effect of different resveratrol concentrations (10-100 µM) IgG concentration (grey) and relative IgG production (qP) (orange) for CHO cells grown in 24-well plates. Each data point represents the average from a quadruplicate, and the error bars correspond to ±SD. * *p*-value < 0.05 for the treatment compared to the control.

The stilbenoid, resveratrol had a considerable effect on CHO cells and the synthesis of IgG. It was tested at 10, 25, 50, 75, and 100 μM (Figure 6). Cell viability remained high between 0 and 50 μM but lost viability at 75 μM. There was a drastic reduction in VCD from 1.22 ± 0.10 × 10^6^ cells per mL at 0 μM to 0.28 ± 0.01 × 10^6^ cells per mL at 50 μM. IgG synthesis also had a slight decline and the calculated *q_p_* rose from 3.2 ± 0.1 pg/cell/day at 0 μM to 5.1 ± 0.2 pg/cell/day at 100 μM. This suggests that resveratrol is of potential use as a cell culture additive where a reduction cell growth is desirable, IgG synthesis is relatively unaffected and an improvement in *q_p_* is desired. Resveratrol has been added to cell culture media before, as a potential inhibitor of autophagy that could improve recombinant production. The work used a single dose of 10 μM resveratrol [23] and observed little effect. Our research indicates a higher concentration of resveratrol is required for a measurable impact on CHO cell growth and IgG synthesis. Resveratrol is also an attractive candidate as an anti-oxidant. Resveratrol can be radicalised at each of the three hydroxyl groups, the 4′ being the most stable. The structure of resveratrol enables the electron from the free radical to freely move around the molecule and it is able to spread the energy across entire molecule providing good stabilisation [24].

**Figure A5:**
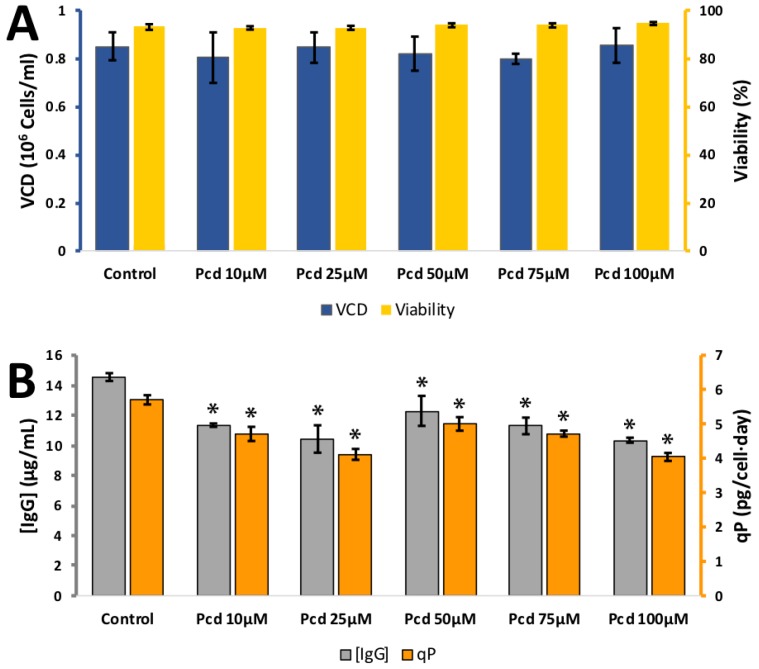
**A**: Effect of different piceid (Pcd) concentrations on viable cell density (VCD) (blue) and viability (yellow). **B**: effect of different piceid concentrations (10–100 µM) IgG concentration (grey) and relative IgG production (qP) (orange) for CHO cells grown in 24-well plates. Each data point represents the average from a quadruplicate, and the error bars correspond to ±SD. * *p*-value < 0.05 for the treatment compared to the control.

Piceid (also known as polydatin) is the glycoside form of resveratrol. It was tested at 10, 25, 50, 75, and 100 μM (Figure A5). Piceid had little effect in viability or VCD, but did reduce IgG synthesis and the resulting low *q_p_* value suggesting picead is a poor candidate for a cell culture additive.

**Figure 7:**
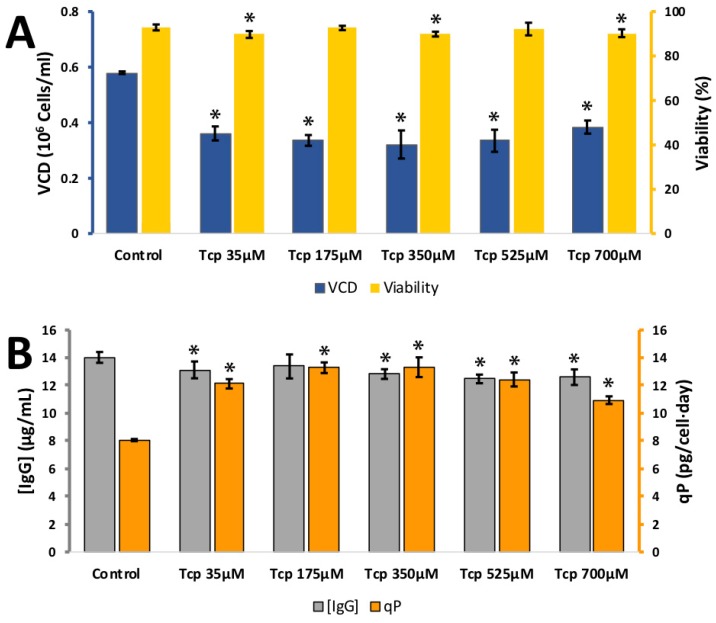
**A**: Effect of different α-tocopherol (Tcp) concentrations on viable cell density (VCD) (blue) and viability (yellow) **B**: effect of different α-tocopherol concentrations (10–100 µM) IgG concentration (grey) and relative IgG production (qP) (orange) for CHO cells grown in 24-well plates. Each data point represents the average from a quadruplicate, and the error bars correspond to ±SD. * *p*-value < 0.05 for the treatment compared to the control.

α-tocopherol is the form of vitamin E used in vitamin tablets. It was tested at 35, 175, 350, 525, and 700 μM (Figure 7). These concentrations were selected to stay under the critical micelle concentration. Cell viability remained high over the whole concentration range. The VCD dropped from 0.58 ± 0.01 × 10^6^ cells per mL at 0 μM to 0.36 ± 0.03 × 10^6^ cells per mL at 35 μM. The IgG synthesis remained stable so the calculated *q_p_* rose from 8.1 ± 0.1 pg/cell/day at 0 μM to 12.1 ± 0.3 pg/cell/day at 35 μM. Higher concentrations of α-tocopherol had no further effect on IgG synthesis or the calculated *q_p_* value. This suggests that α-tocopherol is of potential use as a cell culture additive where a reduction cell growth is desirable, IgG synthesis is relatively unaffected and an improvement in *q_p_* is desired. It is also of added value if its anti-oxidant properties are required as relatively high concentrations can be added to cell culture media (700 μM) without a detrimental effect on cell viability.

4-phenylbutyrate, and glycine betaine also were added to cell culture media to provide a comparison to the polyphenolic screening results (see Figure A2 and Figure A3 in the Appendix A). Interestingly, none of these “chemical chaperones” showed potential as a cell culture additive in this study. Glycine betaine resulted in a slight rise in VCD, but no corresponding rise in IgG synthesis. 4-phenylbutyrate was toxic above 2 mM. At sub-toxic concentrations, it decreased both VCD and IgG synthesis, but with no corresponding rise in *q_p_*. The behaviour of 4-phenylbutyrate observed here is in agreement with previous experimentation where it was added to act as a “chemical chaperone” and reduce aggregation of a misfolded protein [12]. Caffeine was also tested as it has known physiological properties but demonstrated no beneficial effects on CHO cell growth or IgG synthesis (see Figure A4 in the Appendix A).

The screen was repeated and despite variation in the final VCD and IgG concentration and the resulting calculated *q_p_* values in the wells containing the untreated controls, the trends seen in differences in VCD, IgG and the *q_p_* values were consistent between the two sets of plates, indicating that the effect of the additives on CHO cell growth and IgG synthesis were consistent and that the findings were valid.

## 4. Discussion

The early use of antioxidants in cell culture media aimed at reduction of mortality of Chinese hamster ovary cells by reducing the intracellular concentration of reactive oxygen species [25,26]. The species include superoxide anions, hydrogen peroxide, hydroxyl radicals, and singlet oxygen all of which can react directly with the cells and with the recombinant protein product. In one study, the non-phenolic antioxidants reduced glutathione and l-ascorbic acid 2-phosphate were shown to suppress apoptosis and consequently increase synthesis of tissue plasminogen activator [25]. A range of phenolic antioxidants have also been tested for their capacity to reduce reactive oxygen species in CHO cells [15]. The phenolic antioxidants included apigenin, catechin, chlorogenic acid, daidzein, genistein, hesperetin, melatonin, naringenin, pelargonidin, quercetin, resveratrol, rosmarinic acid and silibinin. Interestingly, only two of the phenolics tested, daidzein and genistein, were toxic to the CHO cells. The experimental work indicated catechin and rosmarinic acid performed best in their cultures reducing build-up of lactate and ammonium, and improving the recombinant protein titre. The impact of the antioxidants on chemical modification of the recombinant product was not tested. The phenolic antioxidants, EGCG and rutin, have been used to reduce acidic species charge variants of recombinant therapeutic proteins produced by CHO cells [14]. Experiments using rosmarinic acid in CHO cell culture media reduced oxidative stress which also reduced protein glycation and formation of acidic charge variants [16].

The results of this paper and the work of others [15,27,28,29] suggest the pallet of phenolic antioxidants that could be used to reduce reactive oxygen species in cell cultures is very broad and could include, but not be confined, to the four flavan-3-ols, luteolin, resveratrol, α-tocopherol, chlorogenic acid, silibinin and rosmarinic acid. The effect of the polyphenols on the CHO cells may be to reduce oxidative stress but they also slowed the cell growth (observed as a drop in VCD and an increase in the *q_p_* value). The cause of the slowing of CHO cell growth is not apparent. Polyphenols interact with other molecules by a combination of hydrophobic interaction, hydrogen bonding [30,31] and potential pi-cation interaction. Polymers of phenolics such as proanthocyanidin (polymer of catechin, epicatechin and their gallic acid esters) can cross link and aggregate proteins [32,33,34]. The monomers reversibly interact with proteins with minimal negative impact on solubility. The effect of phenolics on CHO cells in this screen is likely to be due to a range of non-specific interactions with the proteins and phospholipids on the CHO cell surface. The net effect of the interaction of the phenolics (the flavan-3-ols, luteolin and α-tocopherol) was to slow cell growth, retain high cell viability and retain the capability to synthesise IgG.

The options for cell culture strategies that are currently available are diverse. One strategy is to reduce the growth of the cells while maintaining protein synthesis. Reduction of the bioreactor temperature, increasing the osmotic pressure and the addition of chemicals that interfere with the cell cycles can all slow cell growth and increase *q_p_* values. Cell culture additives that reduce reactive oxygen species include both non-phenolic and phenolic antioxidants. The reduction of reactive oxygen species can benefit the cells directly by reducing oxidative stress on the cells but also protect the recombinant protein product from chemical damage caused by free radicals. The use of fatty acid chemical chaperones (butyric acid, valeric acid, valproic acid and 4-phenylbutyric acid) capacity for reducing aggregation of the recombinant protein product, whether in the cell or in the cell culture media is a separate strategy for improving cell culture operations. The beneficial attributes of phenolic antioxidants is their interaction with CHO cells, and the recombinant product is likely to be a combination of their ability to reduce reactive oxygen species and slow cell growth.

## 5. Conclusions

The unpredicted result from this work was that out of the 11 phenolics tested only one was toxic at low doses (CAPE) and only three had no effect on specific cell productivity (kaempferol, curcumin and piceid). The remaining seven phenolics (the four flavan-3-ols, luteolin, resveratrol and α-tocopherol) are potential cell culture media additives with the capacity to raise specific cell productivity as well as the potential of providing some protection from free radical damage. α-tocopherol is the strongest potential candidate for an additive with anti-oxidant properties, due to its low toxicity even at high concentrations (700 μM), which is close to α-tocopherol’s critical micelle concentration. Resveratrol was the most potent cell growth inhibitor whilst enabling IgG synthesis to continue. Resveratrol also is an effective anti-oxidant making it an attractive candidate for further work. The flavan-3-ols and luteolin are potential candidates for causing cell growth inhibition whilst not supressing IgG synthesis and also providing a degree of anti-oxidant protection.

## Figures and Tables

**Figure A6:**
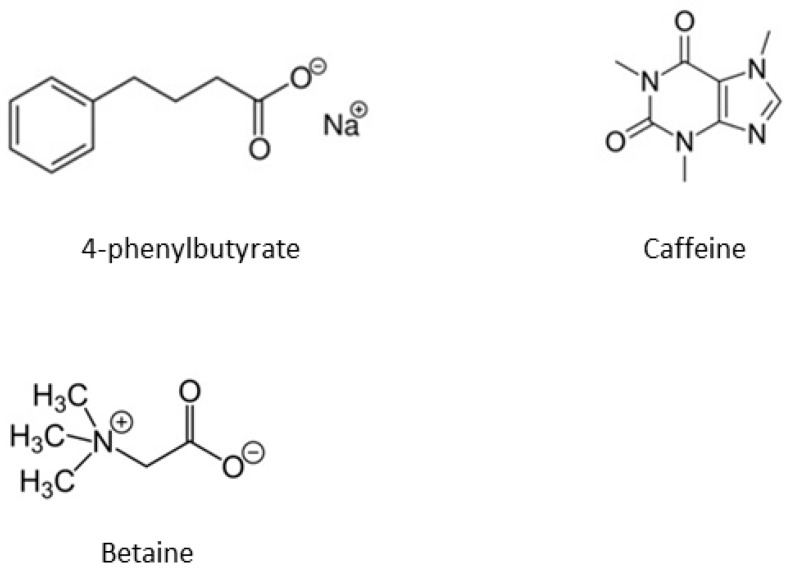
The structures of the “chemical chaperones” 4-phenylbutyrate, the osmolyte glycine betaine and the stimulant caffeine.

**Figure A7:**
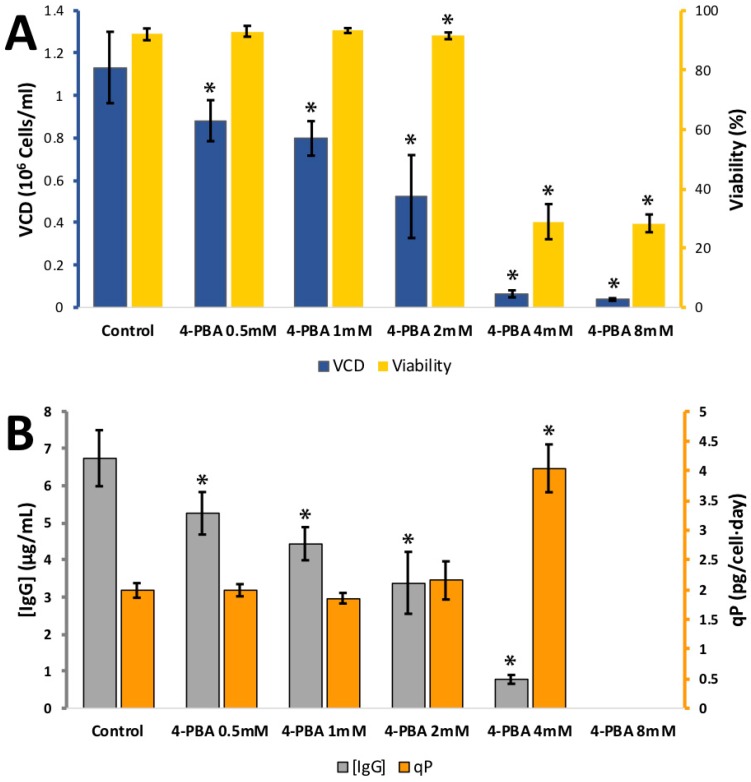
**A**: Effect of different 4-phenylbutyrate (4-PBA) concentrations on viable cell density (VCD) (blue) and viability (yellow). **B**: effect of different α-tocopherol concentrations (10–100 µM) IgG concentration (grey) and relative IgG production (qP) (orange) for CHO cells grown in 24-well plates. Each data point represents the average from a quadruplicate, and the error bars correspond to ±SD. * *p*-value < 0.05 for the treatment compared to the control.

**Figure A8:**
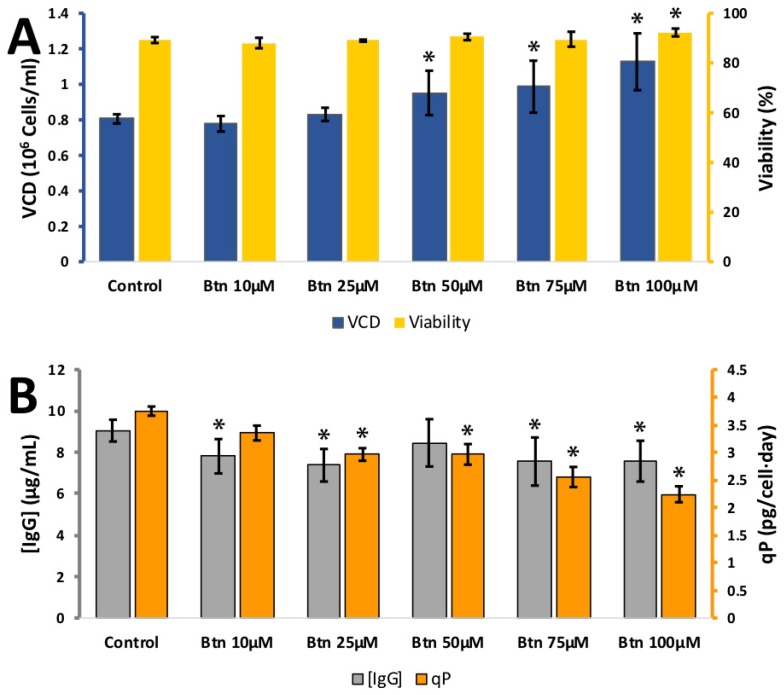
**A**: Effect of different glycine betaine (BTN) concentrations on viable cell density (VCD) (blue) and viability (yellow). **B**: effect of different α-tocopherol concentrations (10–100 µM) IgG concentration (grey) and relative IgG production (qP) (orange) for CHO cells grown in 24-well plates. Each data point represents the average from a quadruplicate, and the error bars correspond to ±SD. * *p*-value < 0.05 for the treatment compared to the control.

**Figure A9:**
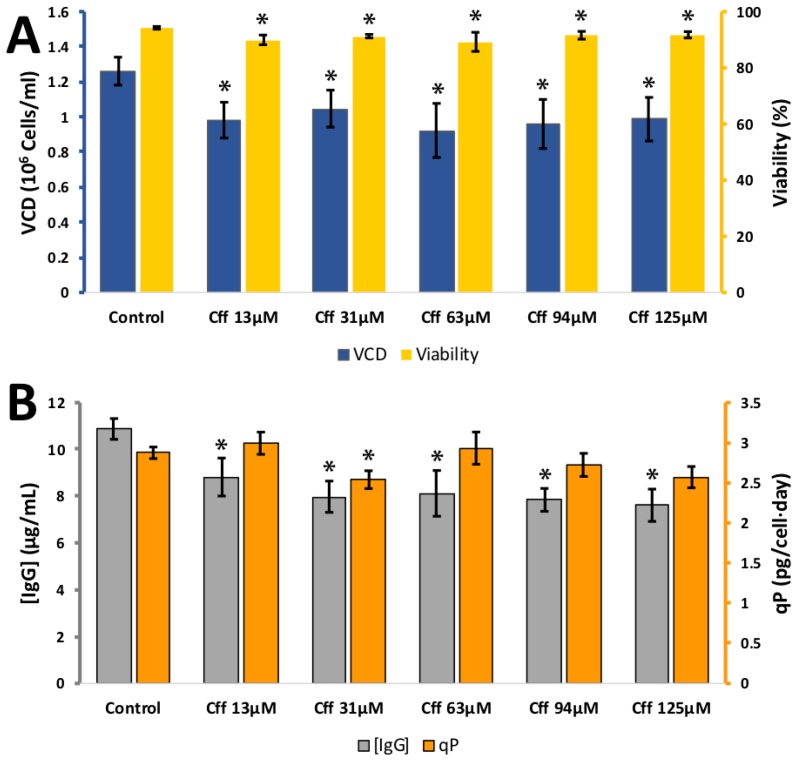
**A**: Effect of different caffeine (Cff) concentrations on viable cell density (VCD) (blue) and viability (yellow). **B**: effect of different α-tocopherol concentrations (10–100 µM) IgG concentration (grey) and relative IgG production (qP) (orange) for CHO cells grown in 24-well plates. Each data point represents the average from a quadruplicate, and the error bars correspond to ±SD. * *p*-value < 0.05 for the treatment compared to the control.
